# Combined Application of Virtual Simulation Technology and 3-Dimensional-Printed Computer-Aided Rapid Prototyping in Autotransplantation of a Mature Third Molar

**DOI:** 10.3390/medicina58070953

**Published:** 2022-07-19

**Authors:** Hui Zhang, Min Cai, Zhiguo Liu, He Liu, Ya Shen, Xiangya Huang

**Affiliations:** 1 Hospital of Stomatology, Guanghua School of Stomatology, Sun Yat-sen University, Guangdong Provincial Key Laboratory of Stomatology, Guangzhou 510055, China; zhhui2@mail.sysu.edu.cn (H.Z.); caim8@mail2.sysu.edu.cn (M.C.); liuzhg6@mail.sysu.edu.cn (Z.L.); 2 Division of Endodontics, Department of Oral Biological & Medical Sciences, Faculty of Dentistry, The University of British Columbia, Vancouver, BC V6T 1Z3, Canada; he.liu@ubc.ca

**Keywords:** autotransplantation of teeth, computer-aided rapid prototyping, virtual simulation, 3-dimensional printing

## Abstract

The use of computer-aided rapid prototyping (CARP) models was considered to reduce surgical trauma and improve outcomes when autotransplantation of teeth (ATT) became a viable alternative for dental rehabilitation. However, ATT is considered technique-sensitive due to its series of complicated surgical procedures and unfavorable outcomes in complex cases. This study reported a novel autotransplantation technique of a 28-year-old patient with an unrestorable lower first molar (#36) with double roots. Regardless of a large shape deviation, a lower third molar (#38) with a completely single root formation was used as the donor tooth. ATT was performed with a combined use of virtual simulation, CARP model-based rehearsed surgery, and tooth replica-guided surgery. A 3D virtual model of the donor and recipient site was generated from cone-beam computed tomographic (CBCT) radiographs prior to surgery for direct virtual superimposition simulation and CARP model fabrication. The virtual simulation indicated that it was necessary to retain cervical alveolar bone during the surgical socket preparation, and an intensive surgical rehearsal was performed on the CARP models. The donor tooth replica was used during the procedure to guide precise socket preparation and avoid periodontal ligament injury. Without an additional fitting trial and extra-alveolar storage, the donor tooth settled naturally into the recipient socket within 30 s. The transplanted tooth showed excellent stability and received routine root canal treatment three weeks post-surgery, and the one-year follow-up examination verified the PDL healing outcome and normal functioning. Patient was satisfied with the transplanted tooth. This cutting-edge technology combines virtual simulation, digital surgery planning, and guided surgery implementation to ensure predictable and minimally invasive therapy in complex cases.

## 1. Introduction

Autotransplantation of teeth (ATT) has emerged as a new alternative for dental rehabilitation, involving use of surgical procedures to replace a deteriorated tooth with a donor tooth transplanted to the recipient site in the same individual [1,2,3]. The survival rate of ATT has been widely reported, with that of immature teeth at approximately 90% [2] and that of mature teeth varying from 59 to 81% [4]. The presence of an intact periodontal ligament (PDL) on the donor tooth, whether mature or immature, is crucial to the outcome of ATT [5]. However, the donor tooth must be used as a surgical guide to prepare the recipient socket, and irreversible damage to the PDL of the donor tooth cannot be avoided during this process.

With recent advances in 3-dimensional (3D) printing technology in dentistry, the application of computer-aided rapid prototyping (CARP) to create a replica of the donor tooth in ATT has improved outcomes [6,7]. Using the CARP replica tooth to guide surgical preparation can reduce the extra-alveolar time of the viable donor tooth and minimize damage to PDL tissues. Several studies have reported the improvement in survival rates in CARP replica-guided ATT compared with conventional ATT, principally in immature roots [4,8,9].

ATT is considered technique-sensitive because of the series of complicated surgical procedures, including minimally invasive tooth extraction, precise socket preparation, and donor tooth replacement [10,11]. Retaining ample buccal and lingual/palatal bone plates of the recipient site during the operation is necessary to ensure the transplanted tooth is tightly repositioned [5]. However, a large shape discrepancy between the root of the donor tooth and the recipient alveolus will increase the difficulty of achieving atraumatic socket preparation and impede transplant stability.

Virtual simulation technology has been widely utilized in pre-clinical education and treatment planning [12,13], but few studies have reported its application in ATT. The present study aimed to introduce the technology in a direct virtual superimposition simulation and a 3D-printed CARP model-based surgical rehearsal of replica-guided autotransplantation to achieve minimally traumatic surgery. Despite the large shape deviation, a mature lower third molar with single-root shape was transplanted to replace a severely compromised lower first molar with double roots, showing excellent transplant stability, PDL healing, and favorable one-year follow-up.

## 2. Case Report

### 2.1. Clinical Examination and Treatment Planning

**Figure 1:**
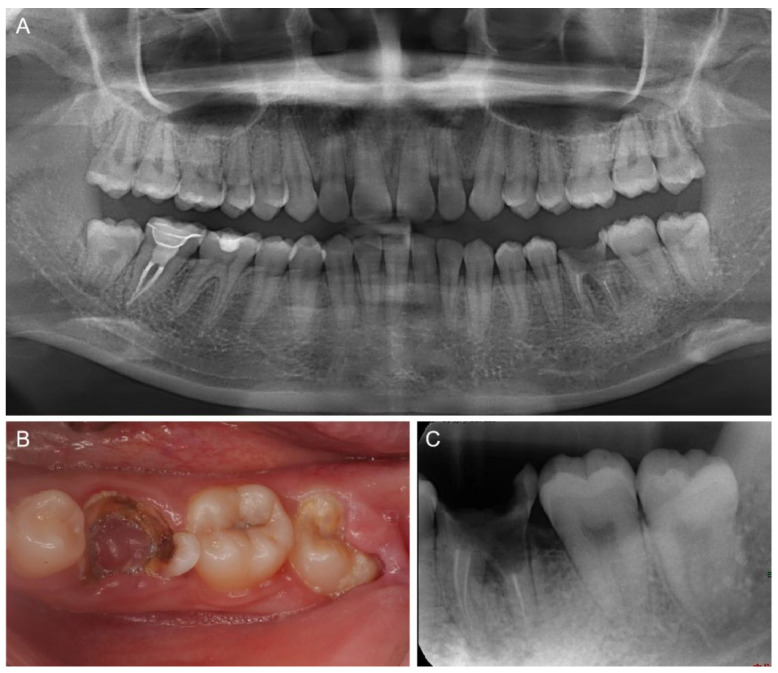
Preoperative examination. (**A**) A panoramic radiograph and (**B**) a clinical photograph showing tooth #36 with extensive caries and pulp polyp, and tooth #38 erupted partly. (**C**) Preoperative periapical radiograph showing tooth #36 with destruction on the floor of the pulp chamber and tooth #38 with completely formed roots.

A 28-year-old female patient presented to the Department of Endodontics at the Hospital of Stomatology (Sun Yat-sen University, China) with the chief concern of a largely destroyed left lower molar. The patient had a history of root canal treatment 5 years earlier. Clinical examination revealed that tooth #36 had extensive caries and pulp polyp, with most of the residual crown below the gingiva. Tooth #38 had erupted partly and showed intact crown structure (Figure 1A,B). A preoperative periapical radiograph showed tooth #36 with destruction on the floor of the pulp chamber and slightly widened PDL space, and tooth #38 with completely formed roots (Figure 1C). Tooth #36 was diagnosed with chronic apical periodontitis and extensive caries; hence, further restoration was hopeless. Having been presented with all the treatment options, including autotransplantation, implantation therapy, and orthodontic treatment, the patient consented to the extraction of the compromised tooth #36, followed by autotransplantation of the mature third molar #38. To maximize the residual alveolar bone and reduce operative time, 3D-printed CARP models of the extraction socket and donor tooth were utilized for preoperative virtual simulation and surgical procedures.

### 2.2. Preoperative Virtual Simulation and Fabrication of the CARP Models

**Figure 2:**
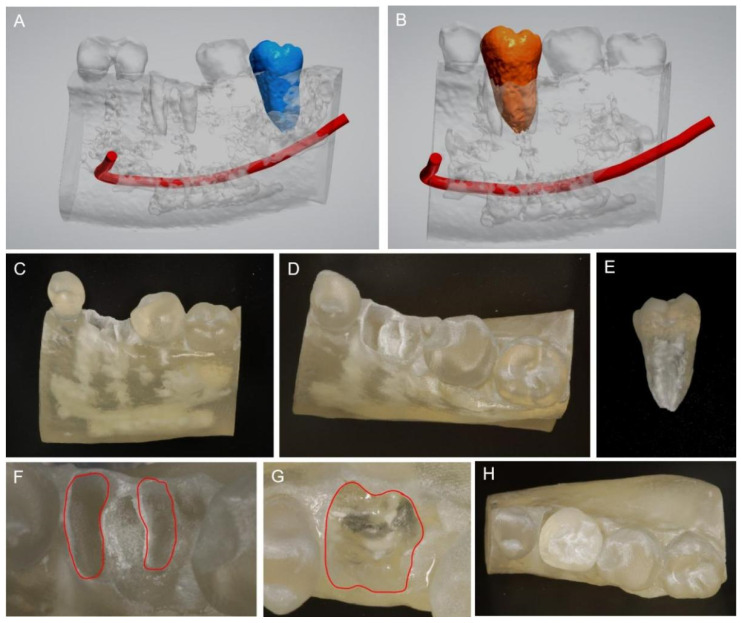
Digital planning and 3D-printed CARP models of the donor tooth and extraction socket. (**A**) Virtual construction of digital imaging was created using CBCT radiographs and surgical planning software (3 Shape Software, Copenhagen, Denmark). Segmentation of the donor tooth #38 (in blue). (**B**) Virtual simulation of the donor tooth in its planned position of tooth #36 (in orange). (**C**,**D**) The 3D-printed CARP model of the tentative extraction socket of the buccal view (**C**) and occlusal view (**D**). (**E**) The 3D-printed CARP model of the replica donor tooth #38. (**F**) Double-root outlines of tooth #36 of the tentative extraction socket are shown as a red line. (**G**) Single-root outline prepared (red line). (**H**) The replica donor tooth #38 inserted into the replica alveolar socket.

The virtual construction of a digital image was created using intra-oral scanning and cone-bean computed tomographic (CBCT) radiographs through surgical planning software (Implant Studio, ver 1.99.1.3.2, 3Shape, Copenhagen, Denmark). Specifically, a threshold value was utilized to generate a mask to isolate the teeth from the surrounding tissues. The segmented teeth were reconstructed and exported into the stereolithography (STL) file [14]. Donor tooth #38 was segmented by blue and the mandibular canal was segmented by red (Figure 2A). The compromised tooth #36 was tentatively trimmed from the 3D virtual images in the 3 Shape software for virtual simulation of the atraumatic extraction socket. Donor tooth #38 was subsequently segmented by orange and placed in its planned position of tooth #36, demonstrating a direct virtual superimposition rehearsal of autotransplantation (Figure 2B). The length of the donor tooth, tentatively trimmed socket, and distance from the transplanted tooth to the mandibular canal could be easily identified via the 3 Shape software. The 3D virtual images revealed the inconsistent root morphology between the donor tooth and recipient tooth, which indicated the difficulty of surgical socket preparation and the risk of low transplant stability.

Thereafter, 3D virtual images of the tentative extraction socket and donor tooth in the software were exported to STL files for fabricating 3D-printed CARP models (NextDent 5100, 3D Systems, Rock Hill, CA, USA). The CARP models of the tentative extraction socket (Figure 2C,D) and donor tooth replica #38 (Figure 2E) were utilized for preoperative rehearsal to shorten the actual preparation time of the recipient alveolar socket. The original double-root outline of the recipient alveolar socket (Figure 2F) was transformed into a single-root outline to accommodate the root contour of the donor tooth replica in vitro, where the maximal buccal and palatal alveolar bone of the recipient socket were retained after socket preparation (Figure 2G). Finally, the donor tooth replica was inserted naturally into the 3D-printed extraction socket of the alveolar bone (Figure 2H).

### 2.3. Clinical Procedures

**Figure 3:**
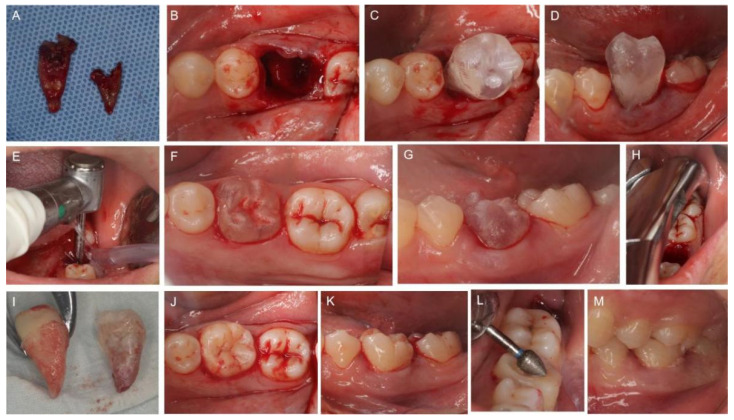
Autotransplantation of tooth #38 into the extraction socket of tooth #36. (**A**) Extracted tooth #36. (**B**) Double root of recipient site. (**C**,**D**) The replica donor tooth #38 is inserted into the extraction socket but does not fit well into the socket in the occlusal view (**C**) and buccal view (**D**). (**E**) Preparation of the recipient site with long neck round bur. (**F**,**G**) The replica tooth fits equitably well into the recipient site in the occlusal view (**F**) and buccal view (**G**). (**H**) Tooth #38 is extracted with minimal trauma. (**I**) Tooth #38 as compared with the replica tooth. (**J**,**K**) The donor tooth #38 is placed into the prepared bone socket immediately and fit well in the occlusal view (**J**) and buccal view (**K**). (**L**) Occlusal adjustment is performed. (**M**) Occlusion is checked at the end of treatment.

Before the surgery, the CARP replica donor tooth was sterilized by high-temperature steam, and the surgical sites were thoroughly cleaned. Autotransplantation of tooth #38 to the extraction socket of tooth #36 was performed under local anesthesia. Minimally traumatic extraction of tooth #36 was first conducted (Figure 3A). The extraction socket was irrigated with 0.9% saline to remove debris. A double-root fossa of the recipient site was observed as the virtual simulation surgery had indicated (Figure 3B). The donor tooth replica #38 was inserted into the extraction socket. As we expected, the replica tooth did not fit well into the socket in the occlusal view and buccal view (Figure 3C,D).

To accommodate the donor tooth in an appropriate space, the recipient socket was subsequently prepared with a long-neck round bur, as had occurred during the in vitro surgical rehearsal on the CARP models (Figure 3E). As indicated by the try-in of the sterilized CARP replica donor tooth into the sockets, the excess alveolar obstacles that impeded tooth insertion were easy to identify and remove. After several attempts at replica tooth try-in and targeted socket preparation, the extraction socket was converted from a double-root shape into a single-root shape within 15 min. Buccal/palatal bone plates surrounding the recipient site were well retained as planned, and the replica tooth #38 settled naturally into the position with good stability (Figure 3F,G).

Since the transplant bed had been successfully created, donor tooth #38 was extracted atraumatically (Figure 3H). Minimally invasive dental forceps were used to reduce the damage to the gingival fibers around the neck of the tooth. An intra-crevicular incision was applied before luxation to reduce PDL injury. No handpiece was used to avoid any damage to PDL tissues. Donor tooth #38 was compared with the replica tooth for the same contour (Figure 3I) and immediately placed into the recipient socket without any extra positioning attempt. The extra-alveolar time of the donor tooth #38 was less than 30 s, and the transplanted tooth fit well with the recipient bone socket in the occlusal view (Figure 3J) and buccal view (Figure 3K). Because of the excellent transplant stability, no rigid fixation was carried out. The transplanted tooth was ensured with infraocclusion by selective grinding to relieve occlusal loading (Figure 3L,M).

## 3. Results

**Figure 4:**
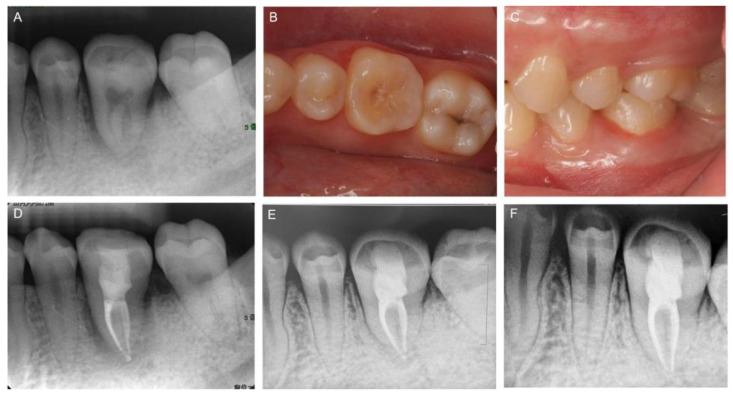
Clinical follow-up. (**A**) Postoperative radiograph taken immediately after autotransplantation. An occlusal view (**B**) and a buccal view (**C**) at the 3-week follow-up. (**D**) Endodontic treatment performed after 3 weeks. The 8-month follow-up radiograph (**E**) and 1-year follow-up radiograph (**F**) showing no signs of root resorption and the normal functioning of the transplanted tooth.

A postoperative radiograph was taken immediately after autotransplantation. The radiolucent region around the donor tooth resulted from the space discrepancy between double root and single root, as expected (Figure 4A). Tooth mobility, occlusion and soft tissue status were checked 3 weeks after autotransplantation (Figure 4B,C), and root canal therapy of the transplanted tooth was also performed in the same visit (Figure 4D). The 8-month follow-up radiograph (Figure 4E) and 1-year follow-up radiograph (Figure 4F) showed no evidence of inflammatory root resorption, ankylosis, or periapical bone lesion. Intact PDL space and lamina dura of the transplanted tooth were also observed. Since the transplanted tooth showed normal functioning compared with other natural teeth, invasive prosthetic restoration was unnecessary. The patient was more than satisfied with the treatment results.

## 4. Discussion

ATT has several intriguing advantages compared to other restoration options. Compared with fixed bridge restoration which requires the preparation of adjacent healthy teeth, ATT is a minimally invasive technique for denture defect restoration [15]. With recent advances in the biological understanding of the periodontal ligament (PDL) healing process, ATT has become an alternative treatment option for nonrestorable teeth [5,11]. Distinct from an osseointegrated dental implant, the viable PDL healing endows the transplanted tooth with physiological mobility, better periodontal margin, and function eruption ability resembling a natural tooth. Hence, ATT has broad indications for adolescents and adults with orthodontic requirements [16]. Mehrangiz et al. reported autotransplantation of tooth #28 with the missing #35 and subsequent orthodontic treatment, which eliminated the need for implants or prosthetic therapy. Normal position of the autotransplanted tooth and good occlusion were achieved after a two-year follow-up [17].

Apart from PDL healing, the optimal success of transplanting the tooth with an immature donor tooth is associated with pulp healing and root formation. Pulp revascularization is possible when the transplanted tooth has immature roots. Favorable PDL healing and pulp healing were statistically significantly associated with the stage of root development. Transplanted teeth with divergent and parallel apical root development exhibited better outcomes than those with convergent roots [18]. It is suggested that donor teeth with immature roots should be at a root development stage higher than stage 4 for appropriate crown–root ratio, regardless of the pulp healing [1]. In the present case, tooth #36 suffered an unmanageable endodontic lesion and the partially erupted mature third molar #38 was an ideal donor tooth for ATT. Compared to tooth #48 with its immature root, tooth #38 with its completely formed root was a better donor candidate because of the same operative region, a consistent inclination of the crown toward the adjacent tooth and appropriate crown–root ratio. In terms of the enlarged surgical trauma and unpredictable root development, the immature tooth #48 (Figure 1A) was not considered the first choice of donor tooth in the current therapy.

Virtual simulation has received much attention in dental education and pre-clinic assessments, such as in implant placement, endodontic microsurgery, orthognathic surgery planning, and virtual prosthetic planning [19,20,21]. Gambarini et al. reported that the dynamic navigation system allowed an undergraduate student to perform planned and guided endodontic surgery by using a virtual patient and computer-aided procedure [21]. However, few studies have reported the application of virtual simulation in ATT. Studies reported that the surgical difficulty and ease of placement of the autotransplant might influence the prognosis of the transplanted tooth [18]. Virtual simulation of ATT will enable easy and predictable surgery. A recent study reported a method of virtual simulation in ATT treatment planning by CARP model fabrication, surgical procedure rehearsal, model rescanning, and computer-aided simulation [4]. The author proposed that this technique enabled the expected osteotomy and immediate repositioning of the immature donor tooth. However, the process of the virtual simulation was time-consuming, and the workflow needed to be simplified, where the CARP models were 3D-printed and rescanned [4].

In the present case, intra-oral scanning and CBCT radiographs of donor and recipient sites were reconstructed directly into the 3D models in the surgical planning software, where the residual roots of tooth #36 in the recipient site were trimmed out to form a tentative extraction socket (Figure 2A). Compared with the previously reported workflow [4], a direct virtual superimposition rehearsal of transferring donor tooth #38 to the tentative extraction socket was subsequently performed, and the overlaid images indicated an extremely large shape deviation in the lower half of the root. This novel technology could not only simplify the workflow but also enhance simulation accuracy by direct superimposition. Moreover, the direct virtual simulation also helps to predict the location of important anatomical structures. As shown in Figure 2B, donor tooth #38 had a single root with a conical shape, while the recipient site had two separated roots. The virtual simulation revealed that the alveolar bone surrounding the cervical area needed to remain for ample bone support. To practice atraumatic socket preparation with maximal buccal and palatal plates retained for the future operation, a surgical rehearsal was successfully conducted on CARP models (Figure 2G).

It has been well documented that the application of CARP models in ATT contributed to a favorable survival rate compared with conventional ATT [4]. The critical factors determining a successful ATT involve the PDL viability and compatible tissue, thus reducing complications such as root resorption and bone loss [11,18]. Hence, unnecessary manipulation of the donor tooth and unrestricted extra-alveolar time (more than 15 min) should be avoided. In this study, the combined application of virtual simulation, rehearsed surgery on CARP models and the replica donor tooth during the operation eventually favored the PDL healing of the transplanted tooth. As the recipient socket was created according to the replica donor tooth, the donor tooth #38 could be placed into the recipient site within 30 s without any additional fitting trials, mechanical trauma or extra-alveolar storage that might damage PDL cells. Wei et al. reported that the success rate and one-year survival rate for computer-aided autotransplantation of teeth were 87.5% and 100%, respectively, with feasible procedures and satisfying clinical accuracy. However, this study only included eight patients [15]. A recent study using computer-aided design combined with 3D printing of the model tooth and surgical guides in ATT also reported similar findings, such as reduced socket preparation time and extra-alveolar time [22]. Despite the advantages of this novel technique, researchers are still concerned about the time-consuming procedures of pre-operative planning and CARP model fabrication. Randomized controlled studies are still required to illustrate the outcome of CARP-based ATT surgery.

Despite the flourishing of 3D printing technology in dentistry, some concerns have also been raised. A significant difference in geometric measurements between the CBCT-generated tooth CARP replicas and the actual tooth was discovered. On average, there was a mean absolute length difference of 0.36 mm and a mean geometric difference of 0.56 mm. Qualitative analysis indicated that the replicas were generally slightly larger in size, which may be beneficial in this clinical context in that the autotransplanted tooth should not be inserted into the recipient site with overpressure [14]. However, if clinically significant changes are found in CARP tooth replicas, excessive bone removal from the recipient alveolus might occur and deteriorate transplant stability.

A donor tooth with a mature root is recommended to complete root canal treatment before or two weeks after surgery [5]. In this case, the impacted donor tooth made it difficult to achieve endodontic access, so endodontic treatment was performed three weeks post-surgery (Figure 4D). Extra-oral RCT during ATT is not recommended because of the risk of PDL injury and prolonged extra-alveolar time [5]. Recent studies stated that only 50% of the patients of CARP-based ATT therapy required a root canal treatment with a 100% autotransplantation survival rate [23,24]. Hence, the studies stated that rapid prototyping-assisted autotransplantation is associated with a reduced RCT rate, but an adequate sample size and long-term follow-ups are required for further study. In the present study, the transplanted donor tooth received RCT therapy and possessed good occlusion, and it achieve a successful outcome at the one-year follow-up.

## 5. Conclusions

This study reported a cutting-edge technique in ATT that combines virtual simulation, digital surgery planning, and guided surgery implementation to ensure predictable and minimally invasive therapy in complex cases. Preoperative virtual simulation and in vitro surgery rehearsal on CARP models were performed to predict and practice the actual autotransplantation surgery. The CARP tooth replica was employed as a surgical guide to create the optimal recipient socket. As a result, the donor tooth settled into the recipient site with an intact PDL and good stability, resulting in a successful PDL healing outcome after 1 year. Future randomized controlled studies on the accuracy and outcome of guided ATT surgery on larger sample sizes are needed.

## Data Availability

Not applicable.
